# Knockdown of BAP31 Downregulates Galectin-3 to Inhibit the Wnt/β-Catenin Signaling Pathway to Modulate 5-FU Chemosensitivity and Cancer Stemness in Colorectal Cancer

**DOI:** 10.3390/ijms241814402

**Published:** 2023-09-21

**Authors:** Jingjing Liu, Qi Zhang, Jiyu Wang, Changli Wang, Tian Lan, Tianyi Wang, Bing Wang

**Affiliations:** Institute of Biochemistry and Molecular Biology, College of Life and Health Sciences, Northeastern University, Shenyang 110819, China; 1910069@stu.neu.edu.cn (J.L.); 1810067@stu.neu.edu.cn (Q.Z.); 2101371@stu.neu.edu.cn (J.W.); 1710070@stu.neu.edu.cn (C.W.); lantian1@mail.neu.edu.cn (T.L.)

**Keywords:** BAP31, 5-fluorouracil, chemosensitivity, stemness, galectin-3, β-catenin

## Abstract

Increased stemness is causally linked to the development of chemoresistance in cancers. B-cell receptor-associated protein 31 (BAP31) has been identified to play an oncogenic role in many types of cancer. However, the role of BAP31 in 5-fluorouracil (5-FU) chemosensitivity and stemness of colorectal cancer (CRC) is still unknown. The aim of this study was to investigate the biological function and molecular mechanism of BAP31 in regulating 5-FU chemosensitivity and stemness. The correlation between BAP31 expression and 5-FU chemosensitivity was examined using 3-(4,5-dimethyl-2-thiazolyl)-2,5-diphenyl-2-H-tetrazolium bromide and colony formation assays. Cancer stemness was analyzed using tumor sphere formation and Western blot assays. Western blot and immunofluorescence analyses of the knockdown cell lines were performed to explore the possible mechanisms. Finally, we investigated the function of BAP31 by constructing xenograft nude mouse models in vivo. In this study, we demonstrated that BAP31 was increased in CRC cells, and knockdown of BAP31 reduced the half maximal inhibitory concentration (IC_50_) of 5-FU, while this effect was reversed by overexpression of BAP31. In addition, knockdown of BAP31 substantially reduced the stemness of CRC cells in vitro. Consistently, knockdown of BAP31 significantly suppressed the tumorigenicity and stemness of CRC in vivo. The functional study further suggested that knockdown of BAP31 downregulated galectin-3 to inhibit the accumulation of β-catenin, which in turn repressed the transcription of downstream target genes (c-MYC, SOX2) of the Wnt/β-catenin signaling pathway. Knockdown of BAP31 reduced stemness by inhibiting the Wnt/β-catenin signaling pathway to increase 5-FU chemosensitivity. Importantly, intrabodies against BAP31 suppressed tumor growth and enhanced the antitumor effects of 5-FU in vivo. Therefore, using intrabodies against BAP31 may be a strategy for improving the antitumor effect of 5-FU in CRC.

## 1. Introduction

Colorectal cancer (CRC) is the most common form of cancer and the fourth-leading cause of cancer-related mortality [1,2]. Treating CRC is challenging; traditional treatment strategies include surgery, chemotherapy, radiotherapy, and targeted therapy [3,4]. Surgery is the basis of treatment for CRC, while chemotherapy is a routine means to improve patients’ prognoses. Most CRC patients are diagnosed at later stages when the cure rate is low due to resurgence and chemotherapy failure. Despite great progress in treating CRC, including the use of 5-fluorouracil (5-FU)-based chemotherapy, the 5-year survival rate is still unsatisfactory [5,6]. Meanwhile, drug resistance is one of the most crucial issues that hinder treatment [7,8]. Therefore, it is necessary to reveal the molecular mechanism of chemotherapy resistance in order to identify more effective therapeutic targets and improve the efficacy of chemotherapy.

Chemoresistance is related to many factors, including increased drug efflux, decreased drug uptake, drug inactivation, phenotypic transformation, defective apoptosis, and target gene mutations [9,10,11,12]. The role of stemness in chemotherapy resistance has been well explained. For example, an analysis of the response to chemotherapy in patients with CRC revealed a close relationship between chemoresistance and stemness gene expression, which appears to be upregulated partly by activation of the stemness program of cancer cells [13]. CRC cells exhibit stemness capability, contributing to the resistance to conventional chemotherapies [14,15]. Therefore, exploring molecules that control CRC stemness will provide therapeutic targets for CRC. It is worth mentioning that an abnormal Wnt/β-catenin signaling pathway is associated with pathological conditions such as cancer. Wnt/β-catenin plays an important role in cancer progression by regulating cell proliferation, metastasis, resistance, and stemness [16,17,18]. β-catenin interacts with transcription factors and regulates the expression of a wide range of Wnt downstream target genes [19]. β-catenin enters the nucleus and binds to TCF transcription factors to activate the transcription of downstream genes, promoting stemness and cancer progression [20].

B-cell receptor-associated protein 31 (BAP31), which is a 28 kDa integral membrane protein located on the endoplasmic reticulum membrane, acts as a membrane protein chaperone and quality control factor with broad specificity. BAP31 also acts as a regulatory protein and is involved in several cellular processes: it supports ER and mitochondrial homeostasis, promotes proliferation and migration, plays various roles in neuroinflammation and the immune system, and regulates autophagy and apoptosis [21,22,23,24,25]. In recent years, with the increasing understanding of BAP31 in cancer, there has been renewed interest in its role in cancer regulatory mechanisms. Accumulating studies demonstrate that BAP31 expression is implicated in the development, progression, and metastasis of human cancers [26,27]. For example, Liu et al. reported that BAP31 promotes metastasis in hepatocellular carcinoma [28]. Our previous study revealed that BAP31 regulates Wnt signaling to modulate cell migration in lung cancer [29]. Intrabodies induce gastric cancer cell death by regulating p27 proteasome degradation [30]. Although BAP31 has been shown to play a crucial role in cancer progression, its role in chemosensitivity and stemness is still unexplained. The aim of the present study was to investigate the biological function and molecular mechanism of BAP31 in regulating 5-FU chemosensitivity and stemness. In our previous study, we used a Proteome Profiler Human XL Oncology Array to analyze the relationships between 84 types of tumor-associated antigens and BAP31, and we found that overexpression of BAP31 increased the expression of galectin-3. In addition, activation of the β-catenin pathway by galectin-3 has been reported in multiple cancer types [31,32]. Therefore, we investigated whether knockdown of BAP31 downregulates galectin-3 to inhibit the Wnt/β-catenin pathway and modulate 5-FU chemosensitivity and cancer stemness in CRC cells. Our findings suggest that BAP31 is a potential therapeutic target for improving the treatment response of CRC with chemosensitivity to 5-FU.

## 2. Results

### 2.1. BAP31 Is Increased in CRC Cells and Associated with Chemosensitivity to 5-FU

**Figure 1:**
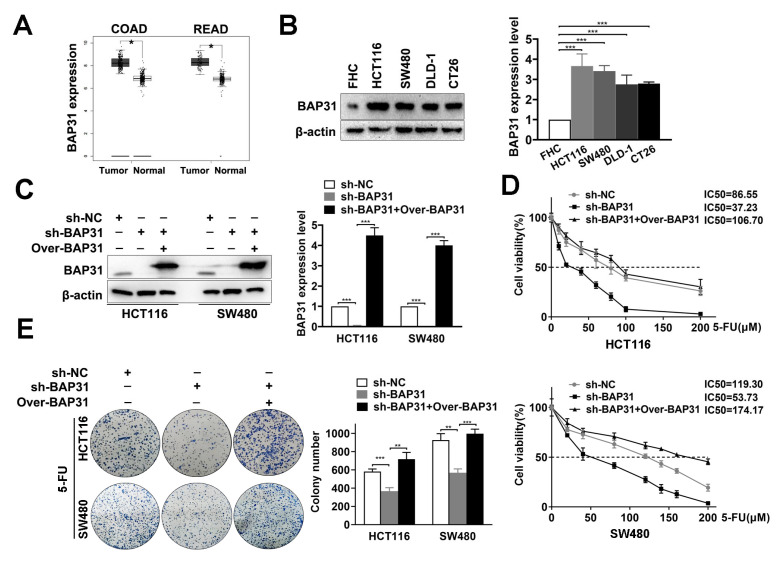
B-cell receptor-associated protein 31 (BAP31) is increased in colorectal cancer (CRC) cells and is associated with chemosensitivity to 5-fluorouracil (5-FU). (**A**) BAP31 levels remained low in normal tissue but were elevated in cancer tissue in a large-scale dataset analysis using GEPIA. (**B**) Western blot analysis was used to detect BAP31 expression in CRC cancer and normal cells. (**C**) Western blot analysis of the BAP31 expression in sh-NC, sh-BAP31, and sh-BAP31+Over-BAP31 cells. (**D**) MTT assay was used to measure viability of HCT116 and SW480 cells treated with different concentrations of 5-FU for 48 h. (**E**) Colony formation ability of HCT116 and SW480 cells treated with 5-FU. β-actin was used as loading control. * *p* < 0.05, ** *p* < 0.01, *** *p* < 0.001.

A large-scale dataset analysis using GEPIA confirmed that the expression of BAP31 is increased in COAD and READ tissue compared with normal tissue (Figure 1A). To validate this observation, we determined the expression of BAP31 in normal and CRC cells. Western blot assay showed that the expression of BAP31 was markedly increased in CRC cells (HCT116, SW480, DLD-1, and CT26) compared with normal cells (FHC) (Figure 1B). Subsequently, to determine the role of BAP31 in 5-FU chemosensitivity in CRC cells, we constructed stable BAP31 knockdown cell lines. HCT116 and SW480 cells were treated with sh-BAP31 (pLKO.1-puro-BAP31) to knock down BAP31 expression and exogenous BAP31 (pc-BAP31-Flag) to increase BAP31 expression. Western blot assay showed that BAP31 expression was significantly reduced in sh-BAP31 cells and significantly increased in sh-BAP31+Over-BAP31 cells (Figure 1C). We then evaluated the chemosensitivity of sh-NC and sh-BAP31 cells to 5-FU. The 3-(4,5-dimethyl-2-thiazolyl)-2,5-diphenyl-2-H-tetrazolium bromide (MTT) assay revealed that knockdown of BAP31 decreased the half maximal inhibitory concentration (IC_50_) of HCT116 and SW480 cells and increased their chemosensitivity to 5-FU. Overexpression of BAP31 in BAP31 knockdown cells increased the IC_50_ and reduced the chemosensitivity of HCT116 and SW480 cells to 5-FU (Figure 1D). Furthermore, colony formation assay revealed that sh-BAP31 cells formed fewer colonies than sh-NC cells in the presence of 5-FU, while this effect was reversed by overexpression of BAP31 in sh-BAP31 cells (Figure 1E). These data suggest that BAP31 is increased in CRC cells and is associated with chemosensitivity to 5-FU.

### 2.2. BAP31 Is Associated with Stemness of CRC Cells In Vitro

**Figure 2:**
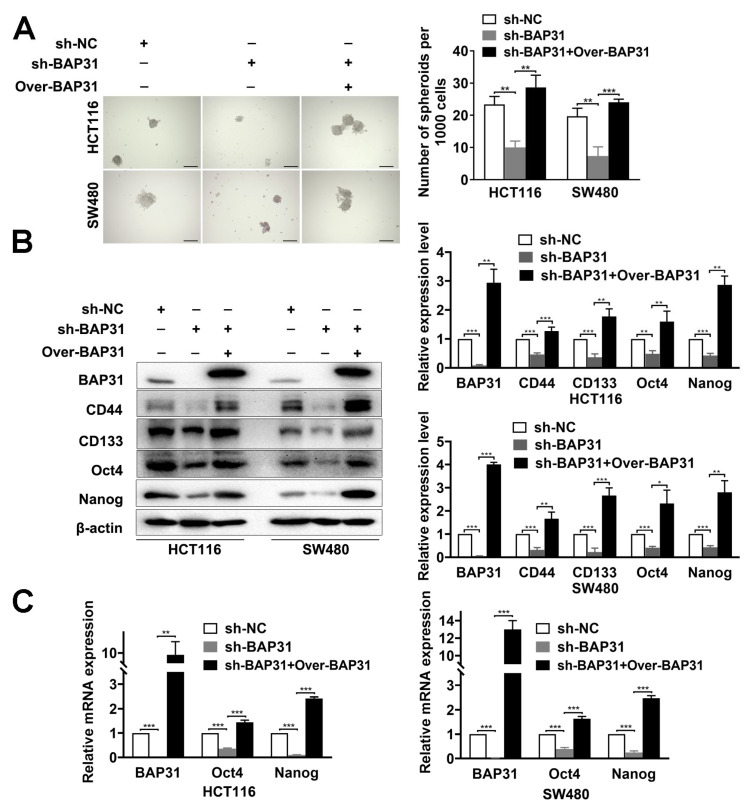
BAP31 is associated with stemness of CRC cells in vitro. (**A**) Representative images of sphere formation assay of HCT116 and SW480 cells. Scale bar, 100 μm. (**B**) Western blot was used to detect expression of BAP31, CD44, CD133, Oct4, and Nanog in HCT116 and SW480 cells. (**C**) Quantitative real-time polymerase chain reaction assay was used to detect mRNA expression of Oct4 and Nanog in HCT116 and SW480 cells. β-actin was used as loading control. * *p* < 0.05, ** *p* < 0.01, *** *p* < 0.001.

Our previous studies examined the relationship between BAP31 and 84 types of tumor-associated antigens using a Proteome Profiler Human XL Oncology Array, and we found that BAP31 has a positive correlation with galectin-3. It has been shown that galectin-3 induces stemness characteristics and enhances the expression of stemness-related genes [33]. Since cancer stemness is a crucial feature of chemoresistance, we wanted to know whether BAP31 modulates stemness in CRC cells. Sphere formation assay showed that knockdown of BAP31 decreased spheroid formation ability and the number of spheroids in HCT116 and SW480 cells. By contrast, overexpression of BAP31 enhanced spheroid formation ability and the number of spheroids in BAP31 knockdown cells (Figure 2A). Canonical cancer stemness-related markers were then determined by Western blotting and quantitative real-time polymerase chain reaction (qRT-PCR) assays. Western blot results revealed that the expression of stemness-related markers (CD44, CD133, Oct4, and Nanog) was decreased when BAP31 was knocked down in HCT116 and SW480 cells, while this effect was reversed by overexpression of BAP31 in BAP31 knockdown cells (Figure 2B). qRT-PCR assay showed that the expression of stemness-related markers (Oct4 and Nanog) was decreased when BAP31 was knocked down in HCT116 and SW480 cells, while this effect was reversed by overexpression of BAP31 in BAP31 knockdown cells (Figure 2C). In summary, our results suggest that BAP31 expression is positively correlated with stemness in vitro.

### 2.3. Knockdown of BAP31 Suppresses Tumorigenesis and Stemness of CRC Cells In Vivo

**Figure 3:**
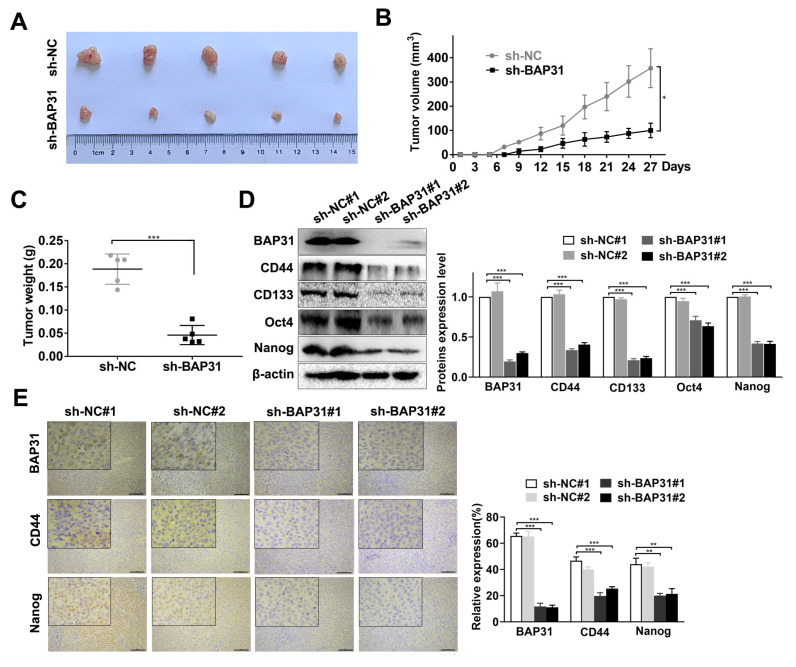
Knockdown of BAP31 suppresses tumorigenesis and stemness of CRC cells in vivo. (**A**) Tumors were harvested and photographed. (**B**) Growth curves of each group of tumors. The indicated numbers of HCT116 cells were inoculated into nude mice and tumor volume was measured every 3 days. (**C**) Each group of tumors was removed and weighed. (**D**) Western blot was used to detect expression of CD44, CD133, Oct4, and Nanog in mouse xenografts. (**E**) BAP31, CD44, and Nanog were evaluated by immunohistochemistry (IHC) in mouse xenografts. Scale bar, 100 μm. β-actin was used as loading control. * *p* < 0.05, ** *p* < 0.01, *** *p* < 0.001.

To validate the function of BAP31 in vivo, we constructed stable BAP31 knockdown HCT116 cells, subcutaneously injected the cells into BALB/c nude mice, and then measured tumor volume and weight in the xenograft mouse models. As shown in Figure 3A–C, knockdown of BAP31 led to a significant reduction in tumor volume and weight compared with the sh-NC group. Next, we examined the expression of stemness-related markers in tumor xenografts by Western blot and immunohistochemistry (IHC) assays. Western blot assay indicated that the expression of CD44, CD133, Oct4, and Nanog was significantly reduced when BAP31 was knocked down (Figure 3D). IHC assay was also performed to validate the results (Figure 3E). These findings indicated that knockdown of BAP31 suppresses CRC tumorigenesis and stemness in vivo.

### 2.4. Knockdown of BAP31 Downregulates Galectin-3 to Inhibit the Wnt/β-Catenin Signaling Pathway

**Figure 4:**
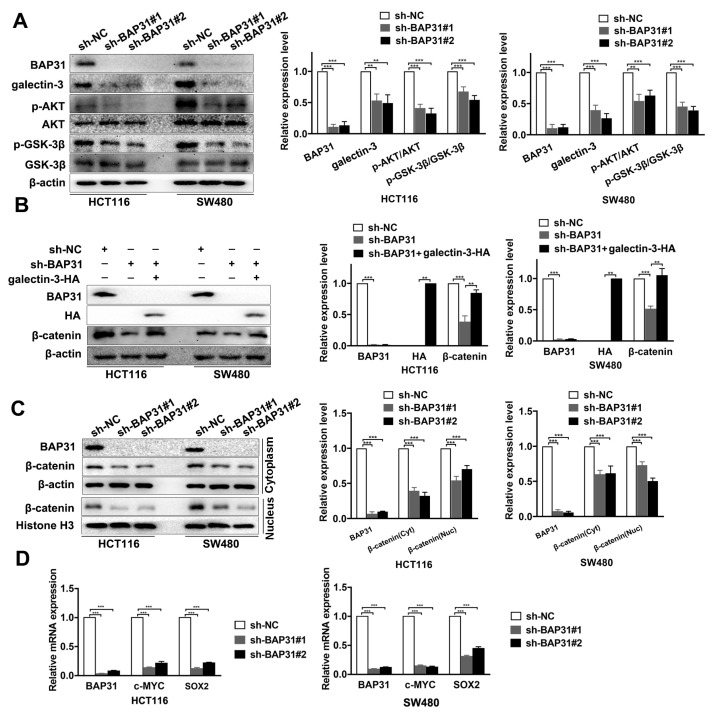
Knockdown of BAP31 downregulates galectin-3 to inhibit Wnt/β-catenin signaling pathway. (**A**) Western blot was used to detect expression of galectin-3, p-AKT/AKT, and p-GSK-3β/ GSK-3β in HCT116 and SW480 cells. (**B**) Western blot was used to detect expression of BAP31, HA, and β-catenin in indicated cancer cells transfected with galectin-3-HA. (**C**) Cytoplasm and nucleus fractions of β-catenin were analyzed by Western blot in HCT116 and SW480 cells. (**D**) Relative mRNA expression of BAP31, c-MYC, and SOX2 in HCT116 and SW480 cells. β-actin and histone H3 were used as loading control. ** *p* < 0.01, *** *p* < 0.001.

Given the positive correlation between BAP31 and galectin-3, we tested the effect of BAP31 on the expression of galectin-3-AKT-GSK-3β signaling. Western blot assay revealed that knockdown of BAP31 downregulated the expression of galectin-3, p-AKT/AKT, and p-GSK-3β/GSK-3β (Figure 4A). Galectin-3 has been reported to play an important role in activating Wnt/β-catenin signaling. To confirm that BAP31 regulates β-catenin through galectin-3, HCT116 and SW480 cells were treated with exogenous galectin-3 (galectin-3-HA) to increase its expression. Western blot results showed that increasing galectin-3 expression did not affect BAP31 expression and rescued the effect of BAP31 knockdown on β-catenin expression (Figure 4B).

Galectin-3 is associated with the nuclear localization of β-catenin, and the Wnt/β-catenin signaling pathway is known to be involved in stemness of CRC [34]. To investigate whether BAP31 regulates the accumulation of β-catenin and activation of β-catenin signaling in HCT116 and SW480 cells, immunofluorescence assay was conducted, which showed that knockdown of BAP31 inhibited the accumulation of β-catenin (Figure S1). Moreover, Western blot assay revealed that knockdown of BAP31 inhibited the accumulation of β-catenin and its translocation to the nucleus in HCT116 and SW480 cells (Figure 4C). In order to determine whether BAP31 regulates the downstream target genes of β-catenin, we evaluated c-MYC and SOX2 mRNA levels. qRT-PCR assay confirmed that knockdown of BAP31 suppresses the transcription of c-MYC and SOX2 in HCT116 and SW480 cells (Figure 4D). The above studies show that knockdown of BAP31 downregulates galectin-3 to inhibit the Wnt/β-catenin signaling pathway, thereby suppressing the transcription of downstream target genes.

### 2.5. BAP31 Regulates Stemness through Wnt/β-Catenin Signaling Pathway

**Figure 5:**
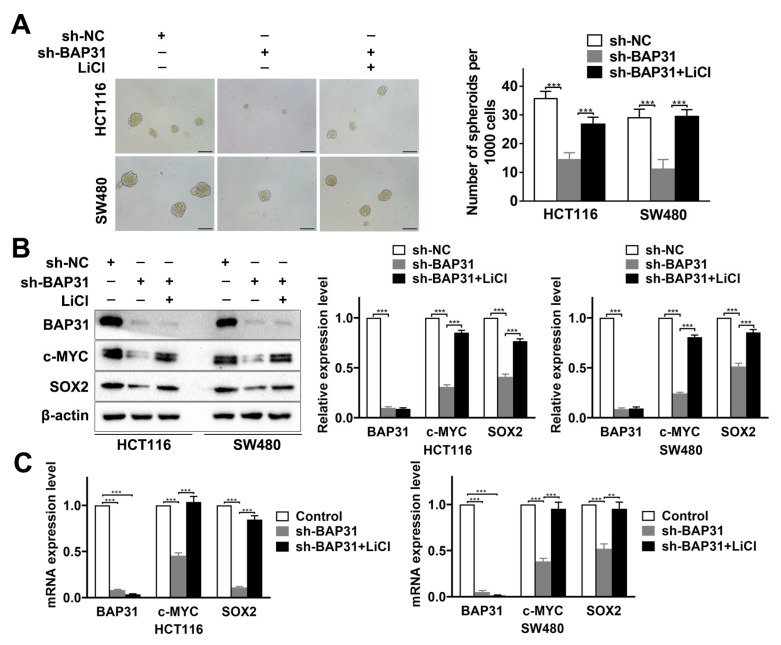
BAP31 regulates stemness through the Wnt/β-catenin signaling pathway. (**A**) Representative images of sphere formation assay of HCT116 and SW480 cells. Scale bar, 100 µm. (**B**) Western blot assay shows expression of BAP31, c-MYC, and SOX2 in HCT116 and SW480 cells treated with LiCl. (**C**) Relative mRNA expression of BAP31, c-MYC, and SOX2 in HCT116 and SW480 cells treated with LiCl. β-actin was used as loading control. ** *p* < 0.01, *** *p* < 0.001.

Based on the above observations, we evaluated whether BAP31 regulates stemness through the Wnt/β-catenin signaling pathway. HCT116 and SW480 cells were treated or not treated with lithium chloride (LiCl, Wnt/β-catenin activator) to determine the effect on BAP31-regulated CRC stemness. Sphere formation assay showed that knockdown of BAP31 decreased spheroid formation ability and the number of spheroids, and LiCl significantly increased spheroid formation ability and the number of spheroids in sh-BAP31 HCT116 and SW480 cells (Figure 5A). Western blot assay showed that knockdown of BAP31 decreased the expression of β-catenin target genes (c-MYC, SOX2), and LiCl upregulated the expression of c-MYC, SOX2 in sh-BAP31 HCT116 and SW480 cells (Figure 5B). qRT-PCR assay was also performed to validate the results (Figure 5C). Thus, it was shown that the activation of β-catenin diminished the inhibitory effects on stemness mediated by the knockdown of BAP31. These results demonstrate that BAP31 regulates stemness in CRC cells through the Wnt/β-catenin signaling pathway.

### 2.6. Knockdown of BAP31 Increases Chemosensitivity to 5-FU by Inhibiting the Wnt/β-Catenin Signaling Pathway

**Figure 6:**
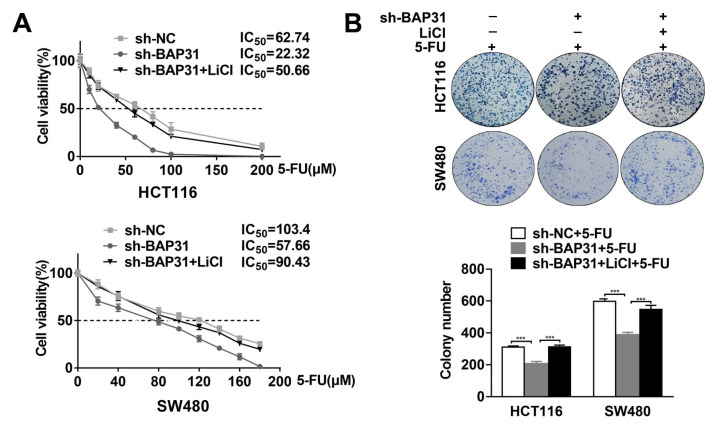
Knockdown of BAP31 increases chemosensitivity to 5-FU by inhibiting the Wnt/β-catenin signaling pathway. (**A**) MTT assay was used to measure the viability of indicated cells treated with 5-FU for 48 h. (**B**) Colony formation ability of indicated cells treated with 5-FU. *** *p* < 0.001.

The Wnt/β-catenin signaling pathway has been identified as a critical regulator in cancer stem cells, leading to tumor progression and chemoresistance. To further test whether the effect of BAP31 on 5-FU chemosensitivity is mainly mediated by the Wnt/β-catenin signaling pathway, we examined whether activation of β-catenin (LiCl) would affect the chemosensitivity to 5-FU of knocked-down BAP31. As shown in Figure 6A, MTT assay revealed that the IC_50_ was reduced by knockdown of BAP31, while this effect was reversed by LiCl. Meanwhile, colony formation ability was reduced by knockdown of BAP31, and this effect was reversed by LiCl in HCT116 and SW480 cells treated with 5-FU, indicating that BAP31 regulates the 5-FU chemosensitivity of CRC cells through β-catenin signaling (Figure 6B). Our results indicate that knockdown of BAP31 increases chemosensitivity to 5-FU in CRC cells via the Wnt/β-catenin signaling pathway.

### 2.7. Intrabodies against BAP31 Enhance Antitumor Effects of 5-FU In Vivo

**Figure 7:**
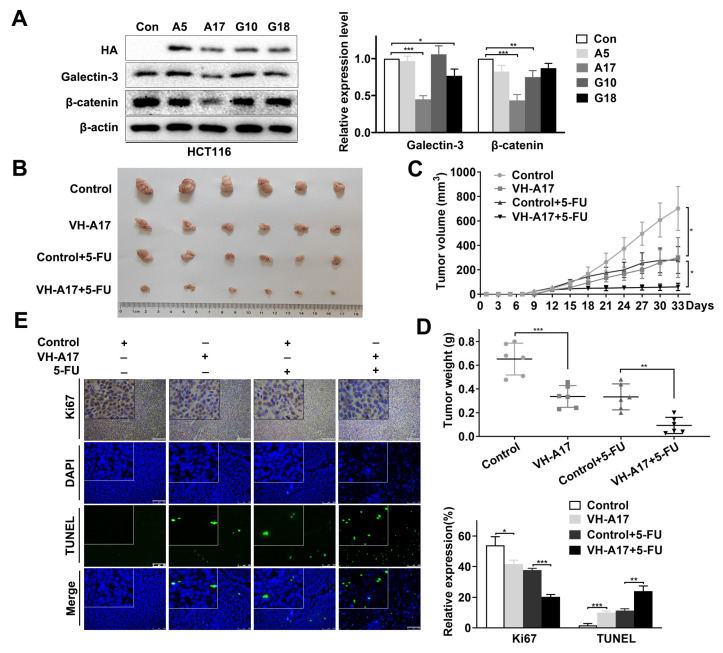
Intrabodies against BAP31 enhance antitumor effects of 5-FU in vivo. (**A**) HCT116 cells were transfected with plasmids containing different VH antibody genes (A5, A17, G10, and G18). Western blot assay was used to detect the effect of VH intrabodies on galectin-3 and β-catenin expression. (**B**) Tumors were harvested and photographed. (**C**) Growth curves of each group of tumors. (**D**) Each group of tumors was removed and weighed. (**E**) Ki67 was evaluated by IHC in mouse xenografts; TUNEL assay of tumor tissue from different treatment groups was performed. Nuclei were stained with DAPI (blue). Scale bar, 100 μm. * *p* < 0.05, ** *p* < 0.01, *** *p* < 0.001.

In our previous study, we screened VH antibodies against BAP31 from a human VH single-domain antibody library [30]. In the current study, we transiently transfected these VH intracellular antibodies into HCT116 cells. Western blot showed that VH-A17 downregulated both galectin-3 and β-catenin expression in HCT116 cells (Figure 7A). Then, the expression of ZsGreen1 after transfection with lentivirus (pLVX-IRES-ZsGreen1-VH-A17) was observed by fluorescence microscopy (Figure S2A), and mice were injected with VH-A17-expressing HCT116 cells to establish CRC xenograft. We investigated the effects of VH-A17 on the mouse organism and assessed the mice by monitoring body weight, organ weight, and organ histomorphology. There were no significant changes in body weight (Figure S2B) or organ weight (Figure S2C). The tissues of heart, lung, liver, and stomach treated with VH-A17 did not show different histomorphology or significant pathological changes (Figure S2D). The results show that the VH-A17-infected cells did not affect the mouse organism.

Next, we examined the effect of VH-A17 on the chemosensitivity of CRC cells to 5-FU. As shown in Figure 7B–D, tumor volume and weight were significantly lower in the VH-A17 group than in the control group, and 5-FU had a better tumor-inhibitory effect in the VH-A17 group. The expression of the proliferation marker Ki67 in tumor sections was detected by IHC staining, and more apoptotic cells were found with 5-FU treatment in the VH-A17 group (Figure 7E). Subsequently, a TdT-mediated dUTP nick-end labeling (TUNEL) assay was used to identify apoptotic cells in tumor tissue. The result showed that extensive apoptosis occurred in the VH-A17 group, and 5-FU treatment further significantly increased the numbers of apoptotic cells (Figure 7E). These data imply that VH-A17 suppresses tumor growth and significantly enhances the antitumor effect of 5-FU in vivo.

## 3. Discussion

The clinical application of 5-FU for treating CRC shows promise [5,6]. However, drug resistance eventually results in treatment failure [35]. In recent years, research has focused on regulating cell stemness and the mechanism of chemoresistance in tumor cells [36,37]. Therefore, inhibiting the formation of stemness may be a new strategy for treating chemoresistance. Studies have shown that BAP31 is involved in many biological processes, including apoptosis [24], invasion [23], and angiogenesis [38]. Despite extensive investigation of the role of BAP31 in cancer, its role in regulating 5-FU chemosensitivity and stemness remains largely unknown.

BAP31 was initially found to be a polytopic integral membrane protein of the endoplasmic reticulum [22]. In recent years, mounting evidence has shown that elevated expression of BAP31 is positively correlated with cancer occurrence and progression in various cancers. For example, inhibiting BAP31 expression inhibits cervical cancer progression by suppressing metastasis and inducing apoptosis [24]. BAP31 promotes migration and invasion in ovarian cancer cells [39]. Meanwhile, BAP31 is overexpressed in CRC tumor tissue, which makes it a potential biomarker for CRC [40,41]. CRC is a leading cause of cancer-related death, with high morbidity and mortality. 5-FU is a chemotherapy drug used to treat malignant tumors, including esophageal, pancreatic, and colorectal cancers [5,42]. The most common treatment method for CRC is to use 5-FU alone or in combination with other therapeutic agents [43]. Chemoresistance of CRC cells plays a pivotal role in cancer treatment, and is closely associated with cancer stemness [8,9]. Therefore, it is crucial to find new targets to reduce resistance. We investigated the role of BAP31 in regulating the chemosensitivity of CRC to 5-FU. In our studies, we discovered that BAP31 was highly expressed in CRC cells. In addition, knocking down BAP31 increased the 5-FU chemosensitivity of CRC cells, while this effect was reversed by overexpression of BAP31. These results suggest that BAP31 is associated with 5-FU chemosensitivity in CRC cells.

We previously examined the relationship between BAP31 and 84 tumor-associated antigens using the Proteome Profiler Human XL Oncology Array and found that BAP31 was positively associated with stemness genes. Stemness is a significant cause of cancer occurrence, development, chemoresistance, and recurrence because it has self-renewal and proliferation abilities [44,45]. Recently, studies have focused on the regulation of stem cell characteristics and the mechanism of drug resistance in a variety of tumors. Research suggests that stemness confers ferroptosis resistance in lung cancer [46]. Wang et al. reported that enhancing the stemness of glioma cells induced temozolomide resistance [47]. Therefore, inhibiting the formation of stemness may be a new strategy for overcoming chemoresistance. Kim et al. proved that BAP31 regulates human embryonic stem cell adhesion, stemness, and survival via control of epithelial cell adhesion molecules [48]. In CRC, markers such as CD133, CD44, Oct-4, and Nanog are commonly used to identify and isolate cancer stem cells [49,50]. In addition, CD133, CD44, and Nanog may have clinical roles in predicting pathological stages, cancer recurrence, and therapy resistance in CRC [51,52]. In this study, the tumor sphere formation assay showed that the expression level of BAP31 was positively correlated with the cancer stemness of CRC. We also discovered that knockdown of BAP31 decreased expression of stemness-related markers such as CD133, CD44, Oct4, and Nanog, while this effect was reversed by overexpression of BAP31. These data suggest that BAP31 might potentially be a factor affecting the stemness of CRC. Najafi et al. reported that CD44 suppression improved the chemosensitivity of HT-29 CRC cells to 5-FU [53]. Khosravi et al. proved that suppression of Nanog increased the sensitivity of CRC cells to 5-FU [54], suggesting that cancer stemness may play an important role in 5-FU chemosensitivity.

Galectin-3 was shown to be a novel binding partner of β-catenin, co-localizing with β-catenin in the nucleus and inducing transcriptional activity of β-catenin target genes [55]. Galectin-3 can also mediate the phosphorylation of AKT, which then promotes GSK-3β phosphorylation and inhibits its activity. Inactivation of GSK-3β leads to the accumulation of β-catenin, which increases nuclear localization and target gene expression [32,33]. In our previous study, we analyzed the relationship between BAP31 and 84 types of tumor-associated antigens using a Proteome Profiler Human XL Oncology Array. We discovered that BAP31 was positively correlated with galectin-3. Galectins play important physiological roles intracellularly [56] and extracellularly [57]. Galectin-3, which can be present intracellularly and extracellularly, is secreted from producing cells and associated with the cells’ plasma membranes [58]. This study focused on how BAP31 controls intracellularly galectin-3 to regulate β-catenin. Our results show that knockdown of BAP31 downregulates the expression of galectin-3, p-AKT/AKT, and p-GSK-3β/GSK-3β. Increasing galectin-3 expression did not affect BAP31 expression and rescued the effect of BAP31 knockdown on β-catenin expression. Therefore, we speculated that knockdown of BAP31 downregulates galectin-3 to regulate the Wnt/β-catenin signaling pathway. It has been suggested that β-catenin is abnormally activated in most CRC patients, making it an important therapeutic target [17,18]. There is accumulating evidence showing that activation of the Wnt/β-catenin signaling pathway may result in nuclear translocation of β-catenin [59]. This suggests that β-catenin may be a downstream signaling target of BAP31. Our subsequent studies further verified this hypothesis: knockdown of BAP31 inhibited the accumulation of β-catenin, which in turn repressed the transcription of downstream target genes of the β-catenin signaling pathway.

Cancer stemness is known to be one of the most important potential mechanisms leading to CRC tumorigenesis and progression [60]. Hence, we asked whether β-catenin is necessary for BAP31 to regulate stemness in CRC cells. Our study found that activating β-catenin rescued the spheroid formation ability and the number of spheroids in BAP31-knockdown CRC cells. Above all, these data suggest that BAP31 regulates stemness through the Wnt/β-catenin signaling pathway of CRC cells. Numerous studies have proved that stem cell therapy may represent an effective treatment for CRC [61,62]. Therefore, eliminating CRC stemness is one of the key strategies for clinical treatment. In our study, BAP31 was found to induce 5-FU chemosensitivity of HCT116 and SW480 cells via the Wnt/β-catenin signaling pathway. We suggest that 5-FU chemoresistance of CRC cells is at least partially mediated by BAP31-induced stemness. Our study indicates that BAP31 is a potential target for CRC treatment with 5-FU, which may provide theoretical clinical support. However, the regulatory mechanism of BAP31 regulating β-catenin is not fully elucidated and needs further exploration.

Targeted cancer therapies are based on monoclonal antibodies and kinase inhibitors, reflecting a new era of cancer therapy. In contrast, delivery to cancer cells is hampered by the large size of conventional antibodies. Nanoantibodies, which are fragments of single-domain antibodies (sdAbs), can overcome this limitation. Human sdAbs consist of a VH or VL variable domain, which has the advantages of low immunogenicity, good solubility, and stability, and is more conducive to therapeutic applications [63,64]. Chen et al. reported that intracellular antibodies targeting HBx suppress invasion and metastasis in hepatitis B virus-related hepatocarcinogenesis [65]. Our previous study found that targeting BAP31 with specific VH intracellular antibodies may be a potential therapeutic strategy with which to inhibit gastric cancer. This study further evaluated the therapeutic effect of VH-A17 on 5-FU for CRC. We used VH-A17 intrabody to verify that intracellular anti-BAP31 could suppress tumor growth and significantly enhance the antitumor effect of 5-FU in vivo. There was no significant toxic response to VH-A17 in mice when observing body weight, organ weight, and organ histomorphometry.

**Figure 8:**
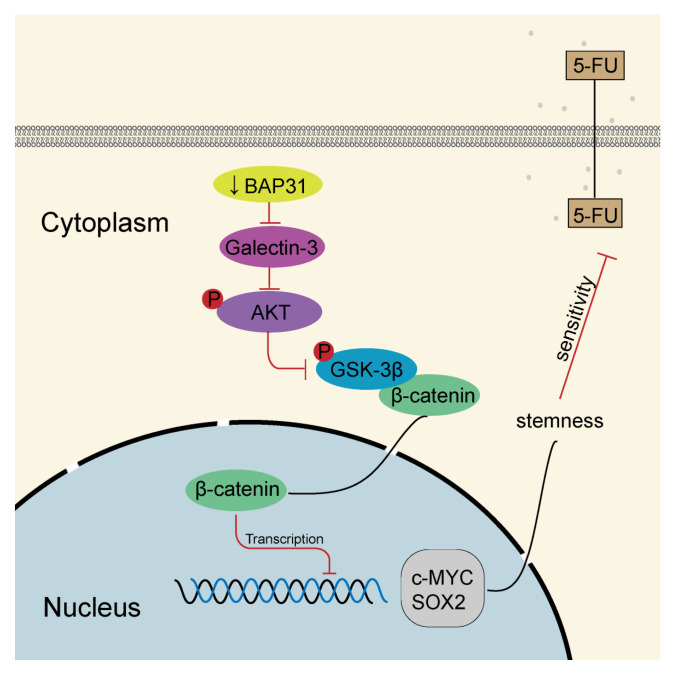
Schematic model of knockdown of BAP31 downregulating galectin-3 to inhibit Wnt/β-catenin signaling pathway to modulate 5-FU chemosensitivity and cancer stemness in colorectal cancer.

The findings of our study are summarized in a schematic diagram (Figure 8). In summary, our research demonstrates that BAP31 is relevant to the 5-FU chemosensitivity and stemness of CRC cells. We elucidate that knockdown of BAP31 increases chemosensitivity to 5-FU by depressing stemness through the downregulation of galectin-3, inhibiting the Wnt/β-catenin pathway. Intrabodies against BAP31 suppress tumor growth and enhance the antitumor effects of 5-FU in vivo. Our findings indicate that targeting BAP31 with a specific VH intrabody might be a potential therapeutic target to improve the treatment response in CRC resistant to 5-FU.

## 4. Materials and Methods

### 4.1. Cell Culture and Chemicals

Two CRC cell lines (HCT116, SW480) were maintained in our laboratory and cultured in DMEM medium (Gibco, New York, NY, USA). All mediums were supplemented with fetal bovine serum, 100 U/mL penicillin, and 0.1 mg/mL streptomycin (Beyotime, Shanghai, China). CRC cells were maintained in 5% CO_2_ at 37 °C incubated with 100% humidity. 5-FU at a purity of ≥99% was purchased from Sigma-Aldrich/Merck KGaA (Darmstadt, Germany).

### 4.2. BAP31 sh-RNA Transfection

To achieve stable and durable BAP31 knockdown in CRC cells, the pLKO.1-puro-shBAP31 lentivirus was constructed and packaged. The lentivirus was packed using 293T cells with two packaging vectors (psPAX2, pMD2.G), and cells were transfected with plasmid using Lipofectamine 2000 (Invitrogen, Carlsbad, CA, USA) according to the manufacturer’s guidelines. After 48 h of infection, the cells were cultured in a medium containing puromycin for 1 month.

### 4.3. MTT and Colony Formation Assays

Cells (2 × 10^3^) were seeded into a 96-well plate, with three replicates set up for each group, and cultured with 5-FU for 48 h. MTT reagent was added to each well, and absorbance at 492 nm was measured after 4 h of incubation on a Synergy H1 microplate reader (Biotek, Burlington, VT, USA). The growth curves of each group of cells were plotted using GraphPad Prism 9 software.

For colony formation, the indicated cells were seeded into a 6-well plate and subsequently treated with 5-FU for 48 h. The medium was replaced every 3 days. After being cultured for 14 days, the colonies were fixed with 4% paraformaldehyde and stained with 0.1% crystal violet (Wanleibio, Shenyang, China). Quantification of colony formation was performed using ImageJ software (V1.8.0).

### 4.4. Tumor Sphere Formation Assay

Cells (1 × 10^2^ cells/mL) were plated in an ultra-low attachment 12-well plate (Corning, Corning, NY, USA) in serum-free DMEM supplemented with B27 (1:50), 20 ng/mL EGF, and 20 ng/mL bFGF. Every 3 days, fresh medium (200 μL) was added. After 14 days, tumor spheres were photographed under a microscope (Leica, Wetzlar, Germany) with a bright field.

### 4.5. qRT-PCR Assay

Total RNA was extracted using Trizol reagent (Sigma-Aldrich, Darmstadt, Germany). cDNA synthesis was performed using a reverse transcription kit (Takara, Dalian, China) according to the manufacturer’s recommendations. Quantitative real-time PCR was performed using 2× SYBR Green qPCR Master Mix (Vazyme, Nanjing, China) on a CFX96 real-time PCR detection system. Relative mRNA expression was calculated as 2-ΔΔCt. The primers (Sangon, Shanghai, China) used are shown in Table 1.

### 4.6. Western Blot Assay

The harvested cells were rinsed with phosphate-buffered saline (PBS) and lysed in radioimmunoprecipitation lysis buffer. The protein concentration was detected using a BCA protein assay (Beyotime). After separation by SDS-PAGE, the protein was transferred onto PVDF membranes (0.22 μm), blocked with 5% nonfat dried milk, and then incubated with primary antibodies overnight at 4 °C. Horseradish peroxidase-conjugated secondary antibodies were used as secondary antibodies. Images were captured using the Bio-Rad ChemiDoc™ imaging system (Bio-Rad, Hercules, CA, USA) and analyzed using Image Lab software (5.2.1). The antibodies used are shown in Table 2.

### 4.7. Immunofluorescence Analysis

The cells were fixed with 4% paraformaldehyde for 30 min and then kept stable in 0.2% Triton X-100 for 30 min to rupture cell membranes. Subsequently, the cells were blocked with 2% bovine serum albumin for 30 min. The cells were then incubated overnight at 4 °C with antibodies against BAP31 antibody (1:200, Abcam, Cambridge, UK) and β-catenin (1:200, Proteintech, Wuhan, China). After washing, the cells were further incubated with the appropriate fluorescence-conjugated secondary antibody (CST, Danvers, MA, USA) for 1 h at room temperature and stained with DAPI (Beyotime). They were then examined by fluorescence microscopy (Leica).

### 4.8. Xenograft Tumors in Nude Mice

For this study, 5-week-old BALB/c nude mice were purchased from SPF Biotechnology Co., Ltd. (Beijing, China), and were maintained in a specific pathogen-free facility. Cells were harvested by trypsinization and resuspended in PBS. PBS (150 μL) containing 6 × 10^6^ cells was inoculated on the right flank of each mouse. Every 3 days after tumor formation, the size was measured, and volume was calculated using the formula L × W^2^/2, where L is length and W is width. 5-FU (20 mg/kg) was prepared with 0.9% NaCl and injected intraperitoneally every 2 days. A 0.9% NaCl solution was used as the control. Tumors were removed 33 days after the mice were inoculated with cancer cells, and tumor volume and weight were compared.

### 4.9. IHC and TUNEL Assays

Tissue sections (5 μm) were deparaffinized in xylene and rehydrated in ethanol. The sections were then treated with peroxidase solution and citrate buffer. Then, after incubation in a blocking buffer for 30 min to block nonspecific binding, sections were incubated with a primary antibody at 4 °C overnight. After being washed with PBS, sections were incubated with secondary antibodies at room temperature for 1 h. After being washed with PBS and stained with horseradish peroxidase (HRP)-conjugated streptavidin for 30 min at room temperature, the sections were developed for 30 s using DAB buffer. Counterstaining was performed with hematoxylin for 5 min, followed by 1 h in running water. Finally, after dehydration, sections were mounted with resinene. Images were observed using an upright microscope (Leica). The primary antibodies were as follows: BAP31 (1:2500, Sigma), CD44 (1:200, Wanleibio), Nanog (1:200, Wanleibio), and Ki67 (1:200, Beyotime).

A TUNEL apoptosis assay kit (Beyotime) was used to detect cell apoptosis according to the manufacturer’s guidelines.

### 4.10. Statistical Analysis

All experimental data were analyzed using SPSS 22.0 statistical software, and a Student’s *t*-test was used to compare the means of two groups of independent samples. Data were presented as the mean ± SD of three independent experiments. Results with *p <* 0.05 were considered statistically significant.

## Figures and Tables

**Table 1:** Primers used in this study.

Primer Name	Primer Sequence (5′→3′)
GAPDH-Forward	GACAGTCAGCCGCATCTTCT
GAPDH-Reverse	TTAAAAGCAGCCCTGGTGAC
BAP31-Forward	CCTCTATGCGGAGGTCTTTGT
BAP31-Reverse	CCGTCACATCATCATACTTCCGA
SOX2-Forward	GCCGAGTGGAAACTTTTGTCG
SOX2-Reverse	GGCAGCGTGTACTTATCCTTCT
Oct4-Forward	CTGGGTTGATCCTCGGACCT
Oct4-Reverse	CCATCGGAGTTGCTCTCCA
Nanog-Forward	AAGCATGTGTTGAACCTCTACC
Nanog-Reverse	TGTGTTGGCTAGTTGGCTTCT
c-MYC-Forward	GGCTCCTGGCAAAAGGTCA
c-MYC-Reverse	CTGCGTAGTTGTGCTGATGT

**Table 2:** Antibodies used in this study.

Antibody	Source	Cat#	Dilution
β-actin	Sigma	A1978	1:10,000
BAP31	Sigma	SAB1406931	1:1000
HA	Sigma	H9658	1:10,000
Histone H3	Abcam	ab308373	1:1000
CD44	Abcam	ab254530	1:2000
CD133	Abcam	ab222782	1:2000
Oct4	Abcam	ab19857	1:1000
SOX2	Abcam	ab97959	1:1000
Nanog	Abcam	ab21624	1:1000
c-MYC	Abcam	ab185656	1:2000
Galectin-3	Abcam	b2785	1:1000
AKT	CST	4685S	1:1000
p-AKT	CST	4060T	1:2000
GSK-3β	Proteintech	67558-1-Ig	1:2000
p-GSK-3β	Proteintech	22104-1-AP	1:2000
β-catenin	Proteintech	51067-2-AP	1:2000
Anti-Mouse IgG-Peroxidase	Sigma	A9044	1:50,000
Anti-Rabbit IgG-Peroxidase	Sigma	A9169	1:50,000

## Data Availability

The data that support the findings of this study are available from the corresponding author upon reasonable request.

## Supplementary Materials

The following supporting information can be downloaded at: https://www.mdpi.com/article/10.3390/ijms241814402/s1.
