# Supplementary material for: Knockdown of BAP31 Downregulates Galectin-3 to Inhibit the Wnt/β-Catenin Signaling Pathway to Modulate 5-FU Chemosensitivity and Cancer Stemness in Colorectal Cancer
Source: Int J Mol Sci. 2023 Sep 21;24(18):14402. doi: 10.3390/ijms241814402 (PMC10532080; doi:10.3390/ijms241814402)
Supplement: Supplementary file 1 [file ijms-24-14402-s001.zip › ijms-2594552-supplementary.pdf]

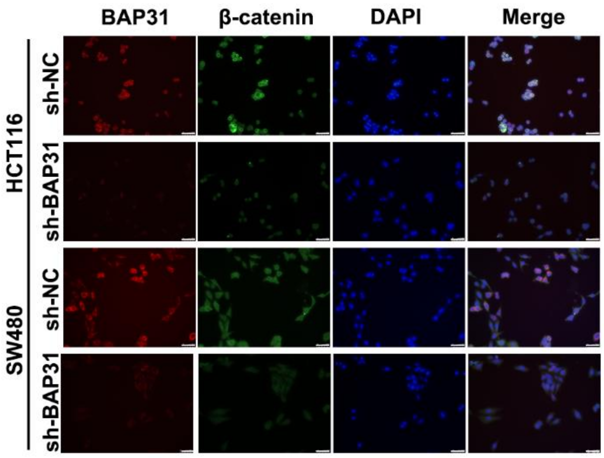

Figure S1. Immunofluorescence determined the distribution of BAP31 (Red) and β-catenin (Green). Scale bars: 250 μm. Nuclei were stained with DAPI (Blue).

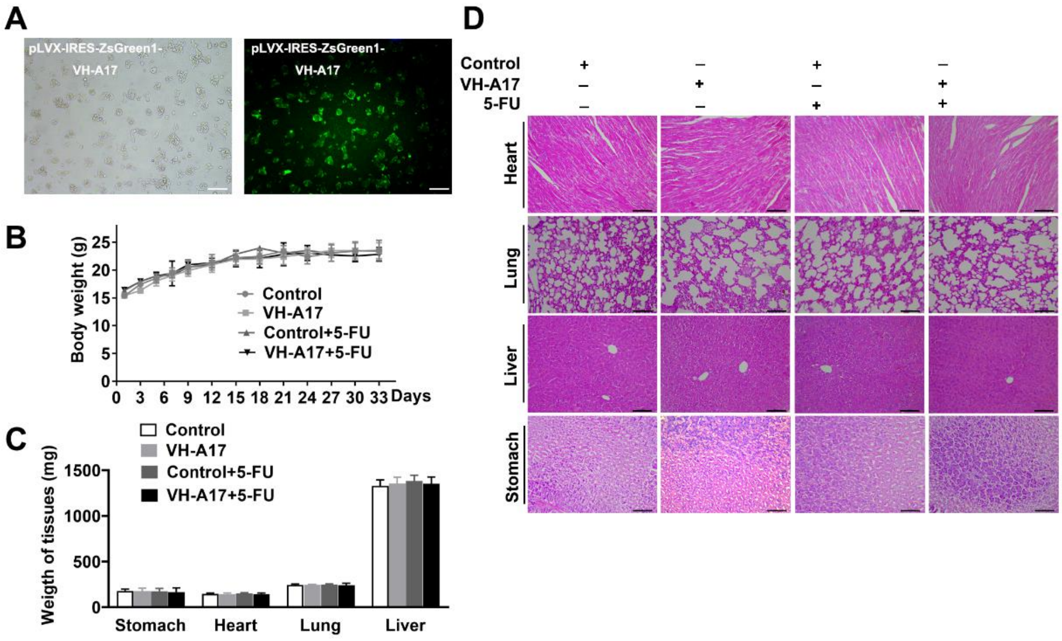

Figure S2. (**A**) The fluorescence of ZsGreen1 in HCT116 cells was observed after 72 h of transduction with lentivirus (pLVX-IRES-ZsGreen1-VH-D1). Scale bars, 100 μm. (**B**) The weight of mice was monitored every three days in the different treatment groups. (**C**) Weight of stomach, heart, liver and lung changes in mice after treatment. (**D**) H&E staining of stomach, lungs, hearts and livers from mice treated with different groups. Scale bars, 100 μm.
